# Analysis of measurement electrode location in bladder urine monitoring using electrical impedance

**DOI:** 10.1186/s12938-019-0651-4

**Published:** 2019-03-22

**Authors:** Yaning Li, Yinglin Peng, Xin Yang, Shipei Lu, Jinwu Gao, Chengguang Lin, Rihui Li

**Affiliations:** 1 Department of Radiation Oncology, Sun Yat-Sen University Cancer Center, 651 Dongfeng East Road, Yuexiu District, Guangzhou, 510060 China; 2 School of Engineering, Sun Yat-Sen University, Guangzhou, China; 3 Department of Biomedical Engineering, University of Houston, 4849 Calhoun Road, Houston, TX 77004 USA

**Keywords:** Computational simulation, Electrical impedance, Urine monitor

## Abstract

**Purpose:**

The aim of this study was to document more appropriate electrode location of a four-electrode-based electrical impedance technology in the monitoring of bladder filling, and to characterize the relationship between bladder filling duration and the measured electrical impedances.

**Methods:**

A simulation study, based on a 2-dimension computational model, was conducted to determine the preferable locations of excitation and measurement electrodes in a conventional four-electrode setup. A human observation study was subsequently performed on eight healthy volunteers during natural bladder urine accumulation to validate the result of the simulation study. The correlation between the bladder filling time and the measured electrical impedance values was evaluated.

**Results:**

The preferable location of measurement electrodes was successively validated by the model simulation study and human observation study. Result obtained via the selected electrodes location revealed a significant negative correlation (*R* = 0.916 ± 0.059, *P* < 0.001) between the measured electrical impedance and the urine accumulation time, which was consistent with the result of simulation study.

**Conclusions:**

The findings in this study not only documented the desirable electrodes location to monitor the process of bladder urine accumulation using four-electrode measurement, but also validated the feasibility of utilizing electrical impedance technique to monitor and estimate the bladder urine volume for those with urological disorders.

## Introduction

Patients suffering from neurological disorders, such as spinal cord injury, generally present a failure of perceiving or voiding their bladders due to neurological damage or muscular atrophy [1, 2]. One common solution to address this problem in clinic is to have a physician or nurse manually inspect the bladder fullness of patients and make sure the patients empty their bladders on time. With the increasing number of patients requiring personal nursing [3, 4], however, the medical and social resources will be consumed heavily [5].

Great efforts have been made to deal with the problem in recent years [6]. Clean intermittent catheterization (CIC) is a conventional approach to drain urine out of bladder by intermittently inserting the catheter in the urethra, though it’s invasive and may possibly result in urinary tract infection [7, 8]. Anatomical imaging technique such as computed tomography (CT), which offers high accuracy in measuring the bladder volume, typically suffers the high radiation, rigorous measurement environment and inappropriateness for continuous monitoring [9]. Ultrasound has been widely employed in clinic to measure the volume of bladder due to its high portability and measurement accuracy [10–13]. Despite these benefits, the ultrasound measurement must be carried out by professionals and is not able to perform continuous monitoring, preventing its use as a routine clinical tool for bladder volume monitoring.

The electrical impedance technique is a developing bio-impedance technology that has been increasingly employed in the estimation and monitoring of bladder volume over the past 30 years [12, 14–17]. The physiological principle of this method relies on hypothesis that the change of bladder urinary volume could induce corresponding change of electrical impedance, which can be measured on body surface [18]. The measurement of bladder volume via electrical impedance technique is typically implemented using a conventional four-electrode method—an alternating current is injected into subject’s lower abdomen through a pair of electrodes attached to the surface, while the induced voltage is simultaneously picked up by another pair of electrodes placed on the surface, the corresponding impedance is consequently computed by the Ohm’s law [19]. It offers multiple benefits over other conventional measurement techniques such as CT and ultrasound; it is noninvasive and features higher portability, lower cost and superior capability for long-term monitoring [20, 21]. These benefits make electrical impedance a potential technique for the real-time and noninvasively monitoring in patients with urological disorder in clinic.

A number of studies have examined the feasibility of utilizing electrical impedance technique to monitor and estimate the bladder volume [16, 18, 22–27]. The results of these projects, including animal and human studies, show that the volume of bladder urine exhibited strong correlation with the surface electrical impedance. Kim et al. applied this method to patients with spinal cord injured and drew the conclusion that electrical impedance has an inverse relationship with the bladder volume [18]. Another study, performed by Shida et al., utilized the four-electrode method to measure the electrical impedance during natural urinary accumulation on a healthy subject [23]. The result suggested that the measured impedance decreased by 7 to 9% during the whole period. Despite these, the electrical impedance is typically sensitive to multiple factors, among which the location of the electrodes is considered as a key role in measuring the electrical impedance [9, 12, 15, 28]. Although multiple studies reported a correlation between bladder volume and the surface electrical impedance, the optimal experimental setup to maximally acquire the physiological information associated with accumulation of bladder urine remains undetermined. It is therefore expected that a more intact study to investigate the effect of various locations of electrodes may help enhance the practical value of electrical impedance in monitoring the bladder volume.

The present study seeks to comprehensively investigate the effect of various locations of electrodes in monitoring the accumulation of urine. To achieve it, a computational simulation study was conducted to determine the desirable locations of excitation and measurement electrodes. A human experiment including eight healthy volunteers was subsequently performed to validate the result of the simulation study and document the relationship between surface electrical impedance and bladder urine volume. We hypothesized that a better correlation between electrical impedance and bladder urinary volume can be obtained using more appropriate location of measurement electrodes.

## Materials and methods

### Simulation model

**Fig. 1:**
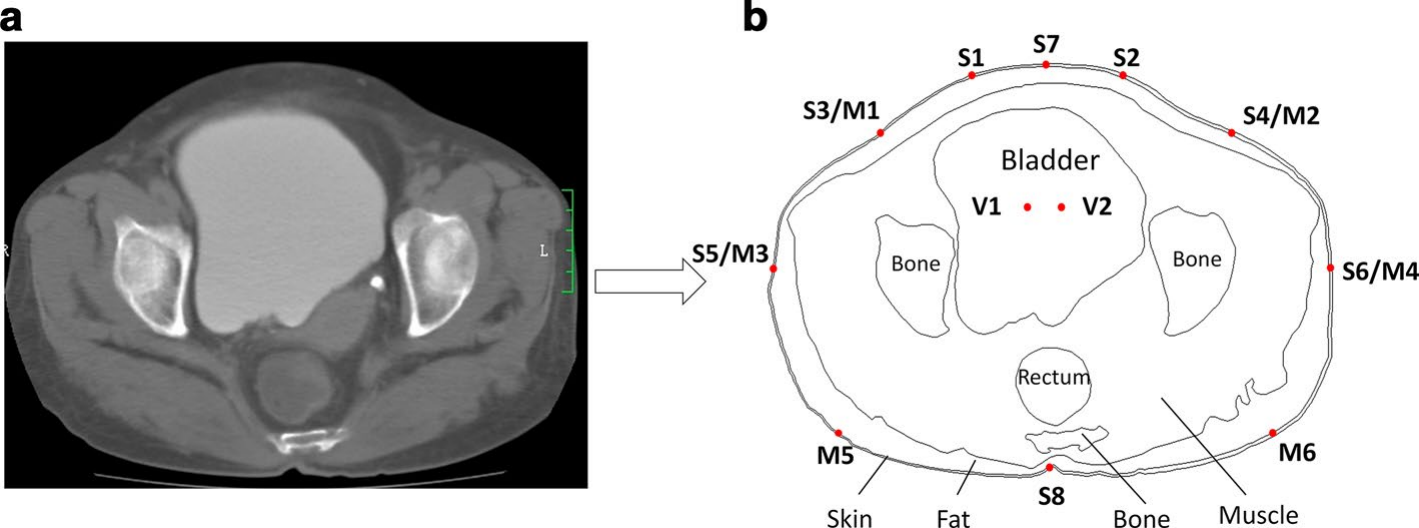
CT image and model used for the simulation study. **a** A CT scan image obtained from a healthy volunteer; **b** 2-D model generated from the CT scan image and locations of all investigated electrodes (S: excitation electrodes; M: measurement electrodes; V: measurement electrodes inside the bladder)

In this study we started with a simulation pelvic model established through finite element method using the COMSOL Multiphysics (4.3a, COMSOL Inc., China). The simulation model contained 15,819 elements was developed based on a computed tomography (CT) scan image of a healthy volunteer, as shown in Fig. 1a. Profile of each component of the human bladder was manually segmented and extracted from the CT image, from which a 2-D pelvic model was built. As shown in Fig. 1b, the model consisted of six kinds of tissues: skin, fat, muscle, bone, bladder and rectum. The electrical properties of these tissues were determined according to literature [29–31], and conductivity of urine in the bladder was set to 1.5 S/m [32], as shown in Table 1.

**Table 1:** The electrical properties (at 10 kHz) of different tissues and fluid used in this study

Tissue	Skin	Fat	Muscle	Bone	Bladder	Rectum	Urine
Conductivity (S/m)	0.00020408	0.02383	0.34083	0.02043	0.21303	0.23995	1.5

The simulation study was then carried out based on a conventional four-electrode configuration with point electrode model. As the location of excitation electrodes seemed to be inconsistent in literature, we first investigated the effects of excitation electrodes at different locations to determine an appropriate location for excitation electrodes. As shown in Fig. 1b, four pairs of excitation electrodes were symmetrically attached to the model boundary (S1–S2, S3–S4, S5–S6 and S7–S8). An alternating current (1 mA p–p; 10 kHz) was injected into the model through the excitation electrodes respectively, and the voltage changes associated with the bladder filling was simultaneously measured by two electrodes placed inside the bladder (V1–V2). It was expected that the preferred location of excitation electrodes gave rise to largest voltage fluctuation during the filling of bladder.

After the excitation electrodes were determined, the ideal location of measurement electrodes was subsequently investigated. As shown in Fig. 1b, an alternating current (1 mA p–p; 10 kHz) was injected into the model through the pre-determined excitation electrodes. The resulted voltages were simultaneously picked up by three pairs of measurement electrodes in various locations (M1–M2, M3–M4 and M5–M6) which represented the front, middle and backside areas of the body. The locations of all placed electrodes are summarized in the Table 2. All measurements were repeated as the bladder volume increased from 0 to 600 mL in increments of 100 mL. In particular, the area of the bladder in Fig. 1b was defined as 600 mL, the change of bladder volume was proportionally scaled relative to the area at 600 mL. The space surrounded the bladder was treated as muscle in the simulation. Data from measurement electrodes at different locations was simultaneously recorded. The optimal location among these measurements electrodes could be subsequently determined.

**Table 2:** The locations of all electrodes in the simulation study

Electrodes	Locations
S1–S2	About 3 cm from the center of abdomen
S3–S4	The midpoint between leftmost point and center of the abdomen; the midpoint between rightmost point and center of the abdomen
S5–S6	The leftmost and rightmost points
S7–S8	The center points of the abdomen and backside
V1–V2	Both sides of the line connecting S7 and S8 (symmetrical)
M1–M2	The midpoint between leftmost point and center of the abdomen; the midpoint between rightmost point and center of the abdomen
M3–M4	The leftmost and rightmost points
M5–M6	The midpoint between leftmost point and center of the backside; the midpoint between rightmost point and center of the backside

### Human experiments

To validate the result of simulation study, a human study was further conducted. Eight healthy volunteers (age: 23–25 years, weight: 55–75 kg, height: 164–178 cm) participated in this experiment. No participant had any history of urological or neurological diseases. The experiment was approved by the ethics committee of Sun Yat-Sen University Cancer Center (Guangzhou, China).

**Fig. 2:**
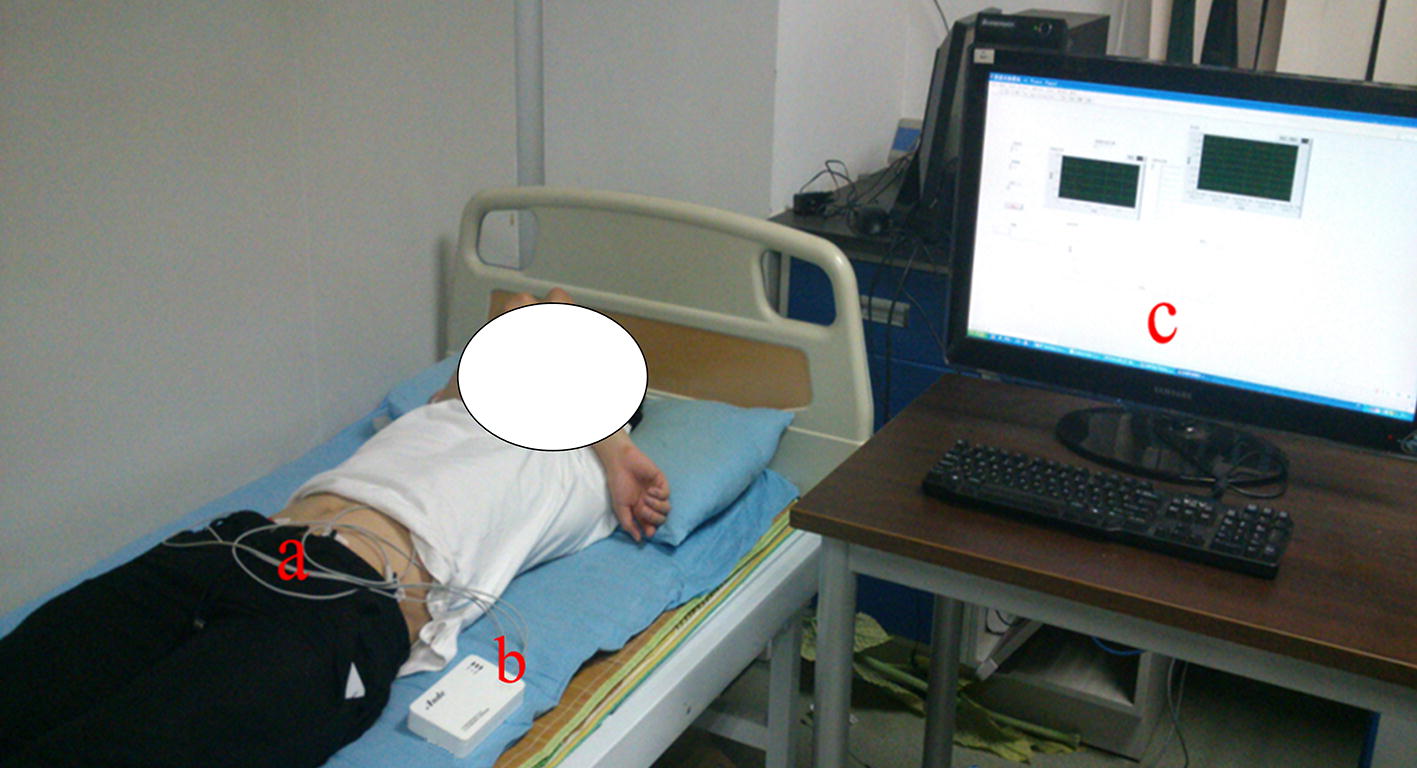
The experimental environment of human study, including healthy volunteers (a), a multichannel electrical impedance measurement system (b) and recording workstation (c)

**Fig. 3:**
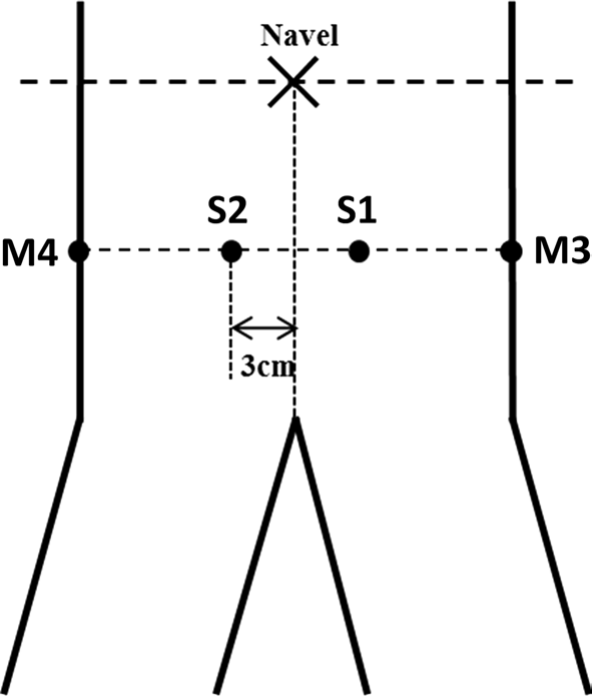
Location of the excitation electrodes (S1 and S2) in human study. The locations of the measurement electrodes (M3 and M4) were changeable

The experiment was performed in a confined room with temperature set to 25 °C and relative humidity between 60 and 70% (Fig. 2). Prior to the experiment, subjects were required to void their bladders, then drink 1000 mL distilled water and lie on a bed. Afterward, eight Ag/AgCl ECG electrodes (2223CN, Minnesota Mining and Manufacturing, USA), including a pair of excitation electrodes and three pairs of measurement electrodes, were attached to their lower abdomen. According to the simulation study, two excitation electrodes (S1–S2) were attached bilaterally 3 cm away from the midpoint between the navel and symphysis pubis (Fig. 3) [18], and the other three pairs of measurement electrodes (M1–M2, M3–M4 and M5–M6) were positioned as shown in Fig. 1. A multichannel electrical impedance measurement system previously developed by our group was employed in this study [15]. During the experiment, an alternating current (1 mA p–p; 10 kHz) was injected into the human body via the excitation electrodes. The induced voltages were measured via each pair of measurement electrodes. As time went by, urine in the bladder accumulated and the voltages of measurement electrodes (M1–M2, M3–M4 and M5–M6) were collected once per 5 min. Subjects were permitted to urinate until they had a strong feeling to void.

### Data analysis

It was expected that the preferable position of excitation and measurement electrodes meets the following criteria. On one hand, the relative change of the measured voltages is strong enough to be captured and processed with modern embedded hardware technology. Secondly, the measured signal should significantly correlate with the filling process of bladder urine.

In this study an indicator named voltage change ratio (VCR) was proposed to quantitatively characterize the change of electrical property associated with the change of bladder volume. The VCR was introduced in a previous study [23] and given as:

1 $$ {\text{VCR = }}\frac{{\left| {V - V_{0} } \right|}}{{V_{0} }} \times 100\% $$

where VCR is the change ratio of measured voltage, *V* is the last measured voltage value, while *V*_0_ is the first measured voltage value. It was expected that the preferable electrodes location revealed a higher VCR when the bladder volume increases from empty to full. Paired *t*-test with Bonferroni multi-comparison correction was performed to test the difference in VCR values between all groups.

In addition, to assess how the measured voltages correlated with the change of bladder volume in human, the Pearson’s correlation coefficient between the measured voltages and the accumulation time of bladder urine was evaluated for all three locations of measurement electrodes.

## Results

### Simulation study

The effect of excitation electrodes with respect to various locations is quantified by VCR values and summarized in Table 3. The VCR at location S1–S2 tended to be higher compared to the other three locations. Therefore, we chose the S1–S2 as a better location of excitation electrodes when investigating the most appropriate location of measurement electrodes in the simulation study and the human study.

**Table 3:** Result summary of the excitation electrodes at different locations

Location	Change of measurement (V)	Voltage change ratio (VCR/%)
S1–S2	2E−05	20.0
S3–S4	3E−05	18.75
S5–S6	2E−05	16.7
S7–S8	1E−05	< 0.01

**Fig. 4:**
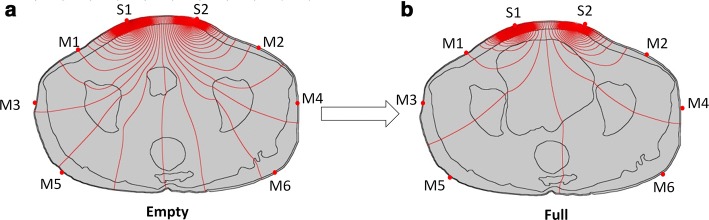
Distribution of isopotential lines (red) from empty bladder (**a**) to full bladder (**b**) in the simulation study

The effect of measurement electrodes at the three locations in simulation study is shown in Fig. 4 and Table 4. It can be observed that isopotential lines changed greater in location M3–M4 and location M5–M6 than that in location M1–M2 as bladder volume changed from empty to full. The VCR values were computed and summarized in Table 4 for each pairs of measurement electrodes (M1–M2, M3–M4 and M5–M6). As shown in Table 4, the measurement electrodes in location M3–M4 gave the highest VCR (63.9%), while the location M1–M2 revealed the lowest VCR (60.5%) value. Therefore, from the simulation study, the location M3–M4 was considered the most desirable position for measurement electrodes.

**Table 4:** Result summary of the measurement electrodes at different locations

Locations	Change of measurement (V)	Voltage change ratio (VCR/%)
M1–M2	7.10E−04	60.5
M3–M4	5.30E−04	63.9
M5–M6	3.20E−04	61.7

### Human study

**Fig. 5:**
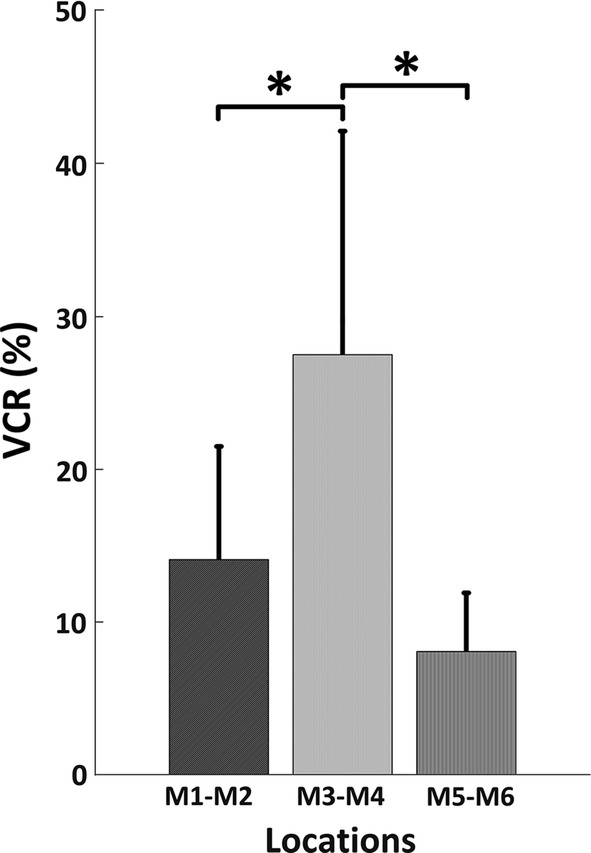
The histogram of the group-averaged VCR for each location. The “*” represents a significant difference between two groups (*p* < 0.05)

The VCR values were computed for each pairs of measurement electrodes (M1–M2, M3–M4 and M5–M6) and summarized in Table 5 over all subjects. It can be observed that the measurement electrodes in location M3–M4 tended to yield the highest mean VCR (27.5 ± 14.7) value over all subjects, while the location M5–M6 gave the lowest mean VCR value (8.1 ± 3.8). Paired *t*-test of the VCR values between location M3–M4 and other two locations demonstrated that VCR values in location M3–M4 was significantly higher than those in other two locations (*p*_*M1*–*M2*–*M3*–*M4*_ = 0.0032, *p*_*M3*–*M4*–*M5*–*M6*_ = 0.0029), as shown in Table 5 and Fig. 5.

**Table 5:** Summary of VCR values and statistical analyses result for all subjects

Subject no.	VCR (%)			Pearson’s correlation coefficient R		
	M1–M2	M3–M4	M5–M6	M1–M2	M3–M4	M5–M6
1	9	26	5	− 0.993	− 0.913	− 0.938
2	4.4	14.3	2.7	− 0.847	− 0.941	− 0.978
3	16.6	23.9	9	− 0.885	− 0.978	− 0.934
4	13.8	16.8	10.6	− 0.927	− 0.942	− 0.978
5	28.6	57.6	12.5	− 0.912	− 0.815	− 0.945
6	18.4	36.1	12.5	− 0.992	− 0.945	− 0.841
7	13.9	32.2	8.3	− 0.920	− 0.835	− 0.939
8	8	13.2	3.9	− 0.962	− 0.958	− 0.977
Mean ± std	14.1 ± 7.5	27.5 ± 14.7	8.1 ± 3.8	− 0.930 ± 0.051	− 0.916 ± 0.059	− 0.941 ± 0.045

**Fig. 6:**
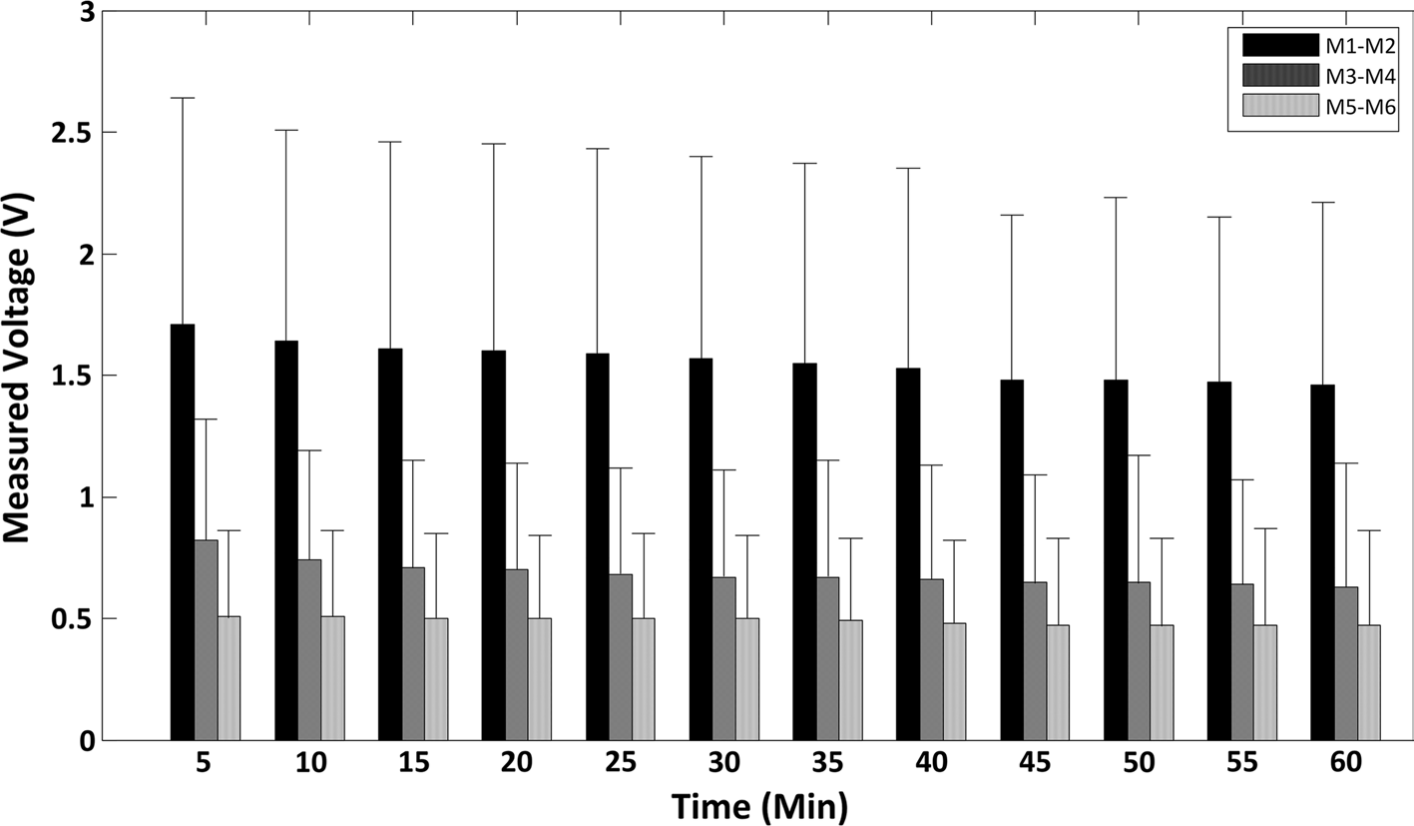
The group-wise summary of the measured voltages for all locations during the urine accumulation process

Figure 6 demonstrates the grand relationship between urine accumulation process (time) and the measured voltages in different measurement locations (M1–M2, M3–M4 and M5–M6) of eight subjects. The Pearson’s correlation coefficient between urine accumulation process and measured voltages for each subject are shown in Table 5. The results clearly indicated a significantly negative correlation between the measured voltages and bladder urine accumulation process in all subjects (*r* < − 0.8), as shown in Fig. 6 and Table 5. In particular, regardless of the locations of measurement electrodes, the measured voltage decreased accordingly as bladder volume increased and statistical analyses through paired *t*-test showed no significant differences between the correlation coefficients of three locations.

## Discussion

Feasibility of utilizing electrical impedance technique to monitoring the accumulation of human bladder volume has been investigated in previous studies [9, 15, 18, 23, 33, 34]. In this study, we attempted to, through a simulation model study and human study, comprehensively explore the preferable location of excitation and measurement electrodes in a four-electrode approach, and to enhance the efficiency of electrical impedance technique in this clinical application.

As demonstrated in previous studies, location of electrodes shows great impact on the sensitivity of electrical impedance measurements [35], which therefore plays a critical role in the measurement of electrical impedance. In this study, through a simulation model as well as a human observation study, we thoroughly evaluated the effects of excitation and measurement electrodes, especially with respect to the locations of electrodes. One specific aim is, we sought to determine a more appropriate location of measurement electrodes that gives rise to the greatest voltage change associated with the filling of bladder urine. In this study the proposed VCR index demonstrated a mean change of 63.9% in simulation study and a mean change of 27.5% in human observation study at location M3–M4, significantly outperforming the VCR values measured from other two locations and results of previous studies (Tables 4, 5) [12, 18]. This finding significantly enhances the sensitivity of the measurement, suggesting a better protocol to maximize the value of electrical impedance in monitoring the accumulation of bladder urine. As such we are able to document a more efficient solution for the monitoring of bladder urine volume using electrical impedance technique.

It is noticeable that although both simulation study and human study suggested the location M3–M4 was preferable compared to other locations, there still apparent difference in the measured VCR values between simulation study and human experiment. In particular, in human experiment the mean VCR at location M3–M4 was only 27.5%, which was not as high as 63.9% in simulation study, as shown in Tables 4 and 5. These differences can be attributed to the following reasons. First of all, compared to the simplified 2D model, the complicated physiological condition within human body has great impact on the measurement of electrical impedance. For example, body fat is one of the most critical factors that affect the measured voltage by coupling electric field around electrodes and subsequently weakening the amplitude of the measured signal. Apart from that, since the pelvic floor is a complicated 3-D structure, the current flows in the pelvic floor will not be fully constraint in the proposed 2-D model, causing a variation in the VCR values between simulated model and human study. It is expect that a comprehensive 3-D model to be developed in the future to allow more precise monitoring of the bladder urinary filling. In addition, though all subjects were required to lie on their back without any movement during the measurement, the position and size of bladders vary from subject to subject, again rendering a variation in the VCR values among subjects. Lastly, urine composition is another factor that might affect the measurement of electrical impedance [18]. It’s known that different kinds of fluids have different conductivity—the compositions of urine are not constant even for one person during the accumulation of urine. Therefore, the measured voltages in the body surface, which is dynamically affected by the conductivity of the fluid in bladder, incorporating the effect of physiological condition of human body, would demonstrate a slight difference compared to simulation study. In our experiment, to minimize this effect, all subjects are not allowed to drink anything except for purified water.

The relationship between electrical impedance and bladder urine volume has been previously explored by several studies [9, 15, 16, 22–25]. As we discussed above, however, it would be challenging to characterize such relation due to various impact factors. In this study, we sought to document this relationship by minimizing the effect of excitation and measurement electrodes—one essential factor in measurement of electrical impedance. It is noteworthy that our experiment results clearly showed a strong negative linear correlation between bladder urine accumulation process and the measured voltages, where the mean value of Person’s correlation coefficient in location M3–M4 achieved − 0.916, as shown in Fig. 6 and Table 5. This result is considered to outperform finding in previous study [18]. Our finding therefore confirms that the monitoring of bladder urine volume using electrical impedance technique is promising. Despite the strong correlation confirmed in this study, however, one apparent limitation is, we are not able to calculate the exact volume of bladder urine by change of electrical impedance during the bladder filling. In our opinion, without taking various impact factors into consideration, such quantitative analysis is insufficient for exact bladder urinary volume estimation in clinical application. However, even we have difficulty in monitoring the exact bladder volume, one meaningful application based on the VCR is to monitor the bladder filling of patients with sensing disorder and to determine when we should remind the patients to void. Such hypothesis was preliminarily tested by a study which reported that the urination point was 7 to 9% decrease of impedance change ratio [23]. Given the small sample size in this study, we will evaluate this potential application on larger cohort of population in the future work.

## Conclusion

The present study aims to document more appropriate location of excitation and measurement electrodes in monitoring the change of electrical impedance associated with the accumulation of urine volume in the bladder. A simulation study was firstly performed to theoretically determine the preferable location of electrodes. Afterward, a human study including eight subjects was conducted to validate the simulation result and characterize the correlation between the bladder urine volume and electrical impedances. In particular, the finding of our study suggests a strong negative correlation between the measured voltages and bladder urine accumulation process, indicating the promising value of utilizing electrical impedance to monitor of bladder urine volume in clinic.
